# A bridge to recovery: an interpretative phenomenological analysis with peer support specialists in Singapore

**DOI:** 10.1080/17482631.2022.2164399

**Published:** 2023-01-19

**Authors:** Jing Ting Lynn Ng, Joanna Barlas

**Affiliations:** School of Social and Health Sciences, James Cook University, Singapore

**Keywords:** Peer support specialist, peer support, recovery, Interpretative Phenomenological Analysis, mental healthcare

## Abstract

Recovery-oriented mental health care approach is gaining acceptance in Asian countries, including Singapore. Following Western countries, Singapore started hiring peer support specialists (PSS) as part of mental healthcare services. The aim of this paper was to explore and understand how individual peer support specialists in Singapore perceive and make sense of their role given their unique perspective as both recipients and providers of mental healthcare treatment. Six PSS in Singapore were interviewed utilizing a semi-structured interview schedule. Interviews were transcribed verbatim and analysed using interpretative phenomenological analysis. Four superordinate themes were generated illustrating how PSS viewed their role: embracing and embodying recovery, balancing on a bridge, impossible without support, and helping to end stigma. Findings also illustrated participants’ awareness of the nature of the job and the role of PSS as still in the infancy stage. They embraced a recovery-oriented mindset despite experiencing stigma from professionals and/or their social support. The need to understand familial attitudes towards the PSS role is discussed. The limitations, contributions to the research, and several areas for future research are also outlined.

## Introduction

Over the last three decades there has been a widespread paradigm shift in mental health services from a traditional biomedical model, which characterizes recovery as an absence or stabilization of symptoms, to a recovery-focused model, which illustrates recovery as an improvement in quality of life (Thorton & Lucas, 2011). Singapore has relatively recently been trying to move towards a recovery approach within its mental healthcare system and has been employing peer support specialists (PSS) since 2012 (Gun & Leong, 2016). PSS are in a unique position of being both recipients and providers of mental healthcare treatment. As such it is important to understand how they perceive and make sense of their role within the mental healthcare system as their perspective sheds light on the ways in which a key tenet of the recovery approach, peer support, is functioning. From a review of the peer-support literature it will be shown that the perspectives of PSS have been sought in several ways but rarely, and never in Singapore, using interpretative phenomenological analysis (IPA), thus providing the foundation for this study.

Clinical recovery comes from a medical model of mental health that views recovery as an absence or stabilization of symptoms and the restoration of premorbid functionality (Slade et al., 2008; Whitley & Drake, 2010). In contrast, the definition of recovery as a transformative process of leading a meaningful life after the diagnosis of mental illness (Chamberlin, 1995; Deegan, 2002) emerged from peer-initiated services which empowered people with mental illness to take charge of their recovery journey during the deinstitutionalization movement and uprising civil rights movement in the United States (Davidson et al., 1999; Slade et al., 2008). Currently, the most widely used and endorsed conceptual framework for recovery (van Weeghel et al., 2019) was generated from a review of 97 articles encompassing descriptions and models of personal recovery (Leamy et al., 2011). The CHIME framework identifies five processes that are deemed to be essential to successful recovery; connectedness, hope and optimism about the future, identity, meaning in life; and empowerment. Andresen et al. (2003) further define five stages of recovery: (i) moratorium; (ii) awareness; (iii) preparation; (iv) rebuilding; and (v) growth.

Western countries are familiar with recovery model approaches and have been including peers to work alongside other non-peer professionals in providing mental healthcare services since deinstitutionalization in the role of PSS (Clossey et al., 2016; Lawton-Smith, 2013). Peer leaders across six continents collectively defined the role of PSS as using their personal recovery experience to guide and facilitate another person’s recovery journey by instilling hope and empowerment to achieve a better quality of life (Stratford et al., 2017).

Since the spread of the recovery model and the increased hiring of PSS, research with PSS has proliferated in several areas, which can be illustrated briefly by consideration of the number of review articles in this field. There are narrative reviews considering the developments, benefits and challenges of PSS (e.g., Davidson et al., 2012; Mahlke et al., 2014; Miyamoto & Sono, 2012; Repper & Carter, 2011), systematic reviews investigating the efficacy and effectiveness of PSS in improving outcomes for individuals with mental health problems (e.g., Ali et al., 2015; Fortuna et al., 2020; Lloyd-Evans et al., 2014; White et al., 2020) as well as facilitators and barriers to implementation (Ibrahim et al., 2020; Mutschler et al., 2021) and a scoping review exploring the mechanisms underpinning peer support work (Watson, 2017).

Within this broad field of PSS research, the experiences of PSS have been studied using qualitative methodologies such as grounded theory (e.g., Clossey et al., 2016; Moran et al., 2013) and interpretative phenomenological analysis (e.g., Dyble et al., 2014); quantitative designs (e.g., Cronise et al., 2016; Lapidos et al., 2018) and mixed-method approaches (e.g., Ahmed et al., 2015; Vandewalle et al., 2016). The existing evidence from these studies indicates both positive and negative experiences in providing peer support, which is further supported and summarized in Walker and Bryant’s (2013) metasynthesis of qualitative findings.

A notable feature of the current research involving PSS is the dominance of samples and settings in Western, and mostly English-speaking countries. For example, across three review papers, a meta-synthesis (Walker & Bryant, 2013), a meta-analysis (Lloyd-Evans et al., 2014) and a systematic review (Mutschler et al., 2021), no studies outside Western countries were included. This is not surprising given that the growth and application of a recovery-oriented approach is still in its initial stages in non-Western countries, including Asian countries (Tse et al., 2013).

At the theoretical level, research into the conceptual understanding of recovery has also tended to be dominated by Western narratives (Leamy et al., 2011; van Weeghel et al., 2019). The field is expanding, however, and a recent scoping review by Kuek et al. (2020) provides an overview of the meaning of recovery in Asia from 30 studies across 10 countries (China, Hong Kong, India, Israel, Indonesia, Japan, Korea, Malaysia, Taiwan). The authors identified dominant themes of recovery from an Asian perspective, including the dual perspectives of symptom remission and successful reintegration into society, cultural and religious explanations of symptoms, and personal, social, and religious factors supporting or hindering recovery.

Research involving PSS in Asia specifically is also limited, and much of the existing research has come from Hong Kong. Qualitative studies have explored the experience of PSS workers in Hong Kong (Tse et al., 2017); how individuals with bipolar disorder made sense of knowledge provided by mental health professionals and peer support workers (Tse et al., 2019); and how family caregivers of individuals with bipolar disorder perceive recovery and peer support workers and how they interact with them (Yuen et al., 2019). Mak et al. (2021) used a longitudinal design to investigate the effect of recovery attributes (e.g., hope, self-esteem) of peer support workers on recovery-related outcomes of mental health service users.

Singapore has been training peers in a Certified Peer Support Specialist Programme since 2016. Currently, the certification course consists of both classroom learning and practicum placement to equip individuals with relevant knowledge and practical skills to provide peer support to peers who are struggling in their own mental health journeys. Participants with a lived experience learn how to share their personal recovery story from a resilient perspective to empower and bring hope to peers, and how to maintain ethical boundaries as a PSS using principles of recovery and resilience (Social Service Institute, 2022). Certified PSSs have been employed by the Institute of Mental Health (IMH) and other mental health voluntary welfare organizations to provide peer support services, which include empowering and/or advocating for clients to the professional staff, providing supportive counselling to clients, sharing strategies to cope with mental illness, and increasing hope for a better quality of life whilst living with a mental health diagnosis (Gun & Leong, 2016).

Public attitude towards mental illness in Singapore is predominantly negative with high levels of prejudice and misconceptions over mental illness in society, as well as reduced tolerance to mental illness, and preference for increasing social distance and restricting social roles for individuals with mental illness (Yuan et al., 2016). Mental health professionals are generally more positive in aspects of tolerance, social restrictiveness, and prejudice and misconceptions but their attitudes towards social distance were similar to that of the general public (Yuan et al., 2017). Therefore, the stigma and discrimination PSS may face in their role may be from the non-peer professionals’ acceptance in the treatment team and possibly from their own families and/or clients’ families.

To date, there has been one published study involving PSS in Singapore; Kuek et al. (2021) used reflexive thematic analysis (Braun & Clarke, 2006) to explore barriers and facilitators of peer support work at IMH in Singapore. Facilitators were identified as supportive figures, having a defined role, opportunities for personal growth and identifying personal coping strategies. Barriers included having an unclear role, hostility from non-PSS staff and unsupportive working environments. The authors hope that their research will “generate more conducive dialogue on changes that need to be made to existing policies and operation frameworks” (Kuek et al., 2021, p. 8) and that more research with PSS in Singapore will help further support these efforts.

Building on Kuek et al.’s (2021) research, this study seeks to understand how individual peer support specialists perceive and make sense of their role within a mental health system that is relatively new to the recovery approach. Being both recipients and providers of treatment within healthcare systems PSS’ narratives offer a window into the unique experience of straddling both sides of healthcare delivery. PSS are both striving towards their own recovery and helping others in their recovery journeys, all within a healthcare system that typically has a medicalized view of recovery. Therefore, asking PSS, as key stakeholders, how they make sense of their role and seeking rich narratives of their lived experiences of providing peer support gets to the heart of the recovery movement and the peer-initiated services it advocates for. The aim of this interpretative phenomenological study is therefore to explore the lived experiences of PSSs and their perceptions of their role working in mental health organizations in Singapore to deepen our understanding of what it is like to provide peer support.

## Method

### Research design

This was an exploratory study, using a qualitative approach and collecting data using in-depth semi-structured interviews, to address the study’s aims. IPA was chosen for its theoretical foundations of phenomenology, hermeneutics, and idiography. IPA was developed to examine personal lived experience and using this methodology allows for convergence and divergence within the participant sample (Smith, 2017). Thematic analysis has been used previously to develop a broad understanding of peer support work across groups of PSS (e.g., Griffiths & Hancock-Johnson, 2017; Kuek et al., 2021; Tse et al., 2019; Yuen et al., 2019). In contrast, IPA has been used with PSS in only one study in the UK (Dyble et al., 2014). Analysing the data collected using IPA allows for a thorough and in-depth exploration of participants’ experiences, which was deemed the most suitable approach to achieve the study aims of exploring the lived experiences of PSS in Singapore to understand how they perceive and make sense of their role within the mental healthcare system. Whilst IPA is an inductive approach, the results will be considered in relation to the CHIME processes (van Weeghel et al., 2019), and stages of recovery (Andresen et al., 2003), as well as Asian perspectives of recovery (Kuek et al., 2020).

### Participants

A purposive sample of six individuals, above 21 years of age, who had worked or were currently working as a PSS for at least 6 months in a mental health organization in Singapore were recruited. This sample size is in line with IPA recommendations for exploring the lived experiences of a homogenous group (Pietkiewicz & Smith, 2017). These six participants reported diagnoses of mood disorders and psychosis, and more information about their work experiences is listed in Table 1. Participants’ ethnic groups were represented by Chinese and Indian individuals. In addition, the educational levels of the participants ranged from GCE “O” Levels to Postgraduate Degrees. Participants were assigned pseudonyms for the duration of the research.

**Table I.** Participant information.

Pseudonym	Gender	Working Experience	Working Status
David	Male	More than 3 years, Worked in more than 1 mental health organization	Full-time, Peer support
Mary	Female	More than 1 year	Full-time, Peer support
Julia	Female	More than 6 months	Part-time, Peer support
John	Male	More than 3 years, Worked in more than 1 mental health organization	Full-time, Peer support
Anna	Female	More than 1 year	Part-time, Peer support
Susan	Female	More than 1 year	Full-time, Integrated job: Peer support and non-peer role

### Procedure

Ethical approval (approval number H7076) was granted by James Cook University Human Research Ethics Committee. Participants were recruited through a contact person who assisted in disseminating information about the study to the pool of PSS in Singapore. Interested participants contacted the first author by email or telephone. Written informed consent was obtained from all participants. Socio-demographic details (e.g., gender, age, ethnicity, and other information such as mental health diagnosis and remuneration) were gathered using a questionnaire.

The first author conducted semi-structured interviews consisting of open-ended questions and prompts to gather in-depth narratives from each participant about their experiences as PSS. The semi-structured interview schedule was developed from literature and refined through discussions between the first and second authors. To understand how PSS view their role it was deemed important to start with an open question about their experiences as a PSS and then three subsequent questions that probed their view of how the role helps in their own recovery, how they make use of support within the role, and how they see the role fitting within the broader system. The interview schedule was applied flexibly, and prompts were used to probe further into participants’ unique experiences in line with IPA’s idiographic approach. The interviews were between 40 and 110 minutes long and were audio recorded and transcribed verbatim, alongside additional notes (e.g., nonverbal gestures) taken by the first author during the interviews.

### Data analysis

IPA was used to analyse the data collected (Smith & Osborn, 2008) with the aim to understand how PSS make sense of and view their role. Following the guidelines of Pietkiewicz and Smith (2014), the initial process of analysis involved the first author familiarizing herself with each transcript and annotating significant comments, similarities and differences, and making preliminary interpretations in the left-hand margin. These initial interpretative comments were centred around participants’ descriptions of their experiences as a window into how they made sense of the different aspects of their role. Next, the first author read through the transcript to build on these provisional notes and interpretations and document emerging themes in the right-hand margin that involved more abstract concepts. The first author listed emergent themes separately for each transcript, looked for connections and clustered similar themes and superordinate concepts that moved beyond that which was stated explicitly in the transcripts. The entire process was then repeated with the remaining transcripts, one-by-one. Once all the transcripts were analysed, the first author looked across all the transcripts for repeated themes to form superordinate themes which provided valuable insights into the participants’ experience as a PSS and enabled us to understand how they view their role. The themes were then checked back against the transcripts to ensure they were grounded within participants’ experiences and Smith’s (2011) guideline of an agreement of at least three cases having the same theme to form the superordinate theme was followed.

The double hermeneutic stance of IPA recognizes that the authors’ professional and personal experiences may lead to preconceptions and biases within the analysis process (Smith, 2011), as such the authors’ backgrounds are disclosed here. The first author is a Singaporean clinical psychologist who was in training at the time of interviewing. The first author has experience working in local mental health services, specifically with PSS, and is supportive of the introduction of a recovery approach to Singapore. The second author (the first author’s research supervisor) is a British lecturer in clinical psychology at an Australian university in Singapore. The second author has previous experience of working in secondary and tertiary mental health services in the United Kingdom. Both authors hold both personal and professional beliefs about the importance of the recovery approach.

Throughout the analysis the first author discussed her annotations and emerging themes with the second author as part of the reflexive process during supervision sessions. This included reflections on preconceptions and biases stemming from previous work and academic experiences. Both qualitative (Yardley, 2000) and IPA specific (Nizza et al., 2021) guidelines were followed to ensure rigour and quality control in the research.

## Results

Four superordinate themes were generated illustrating how PSS made sense of and viewed their role: embracing and embodying recovery, balancing on a bridge, impossible without support, and helping to end stigma (Table 2). The order of the superordinate themes was arranged to illustrate the lived experience of PSS’s view of their role.

**Table II.** Compositional structure of IPA themes.

Superordinate themes	Subordinate themes
1. Embracing and embodying recovery	1.1 Acceptance and opportunity1.2 Recovery is reinforced
2. Balancing on a bridge	2.1 Looking after self versus others2.2 Pushing forward and pulling back
3. Impossible without support	3.1 Family support3.2 Professional support3.3 Faith
4. Helping to end stigma	4.1 Using self as a tool4.2 One voice in the system

### Embracing and embodying recovery

This superordinate theme captured the sense of the PSS role being intrinsic to participants’ own recovery. Through deliberate openness and choice in accepting their own mental health problems they saw the PSS role as offering opportunities to move closer to their own recovery and to help others in their recovery journeys in a mutually beneficial cycle. Their reflective stance enabled them to view their own mental health problems from a position of positivity, and they were grateful that the PSS role allowed them to derive meaning and fulfilment from their own, likely challenging, past experiences. Two subordinate themes were generated.

#### Acceptance and opportunity

Most participants viewed the PSS role as closely intertwined with the ongoing process of accepting their mental health diagnosis and the trajectory of their mental health condition. Participants spoke about the stigma they had experienced associated with their mental health diagnosis from themselves and others within various situations while on their own recovery journey and when providing peer support. Positivity contributed to participants’ level of acceptance of their mental health diagnosis and potential setbacks in their recovery journey. Mary explained, *“ I have accepted this truth as a reality and not living in denial but really face it positively. So, I hope my sharing of my recovery story … they (peers) can walk towards recovery and not give up hope*”. Mary’s experience highlighted her own struggles in accepting her diagnosis *“Because of this mental health condition, I have to give up my previous (non-PSS) job*”, nonetheless, her turning point in her recovery journey was her acceptance. Thereafter, she managed to embark on her own recovery journey and through her role, she was able to help others in their recovery journey *“ …It’s like I am encouraging myself and also encouraging the peers too”*. Similarly, David’s change of perspective about his own mental health condition enabled him to see it as an opportunity for helping others *“…My mental illness seemed to be a curse in the beginning but now it’s a blessing that can share recovery for others ”.*

Half the participants viewed the PSS role from a position of gratitude as it provided them with meaningful and purposeful work opportunities that left them with a sense of fulfilment. In Mary’s narrative, she shared her feelings of gratitude several times *“…grateful that I am doing such a good job”*. For Susan, she felt her role gave her meaning in her life after her mental health diagnosis*“ Recovery also means like for me doing a meaningful job, doing a peer support specialist job is a meaningful and I can contribute back to society and I can live a meaningful life”*. Anna also shared the satisfaction experienced from her role as PSS *“ I found that there’s a lot of fulfilment in peer support specialist because I really get to help others and I really enjoy like helping and being in touch with my own issues and I really enjoy working with people ”*. They seemed to view their own recovery as an ongoing testimony of the approach while bringing about recovery to peers in need.

Additionally, all participants emphasized the importance of approaching the PSS work from a stance of positivity. They remained positive despite challenges from various sources, including peers, caregivers, and the workplace. Adopting a positive mindset appeared to facilitate growth and development in their role. John explained, *“When we become peer support specialists, we have to face the challenges, and we see the challenges as not as problem, not as a roadblock, but as obstacles, which we can eventually overcome to grow and develop ourselves”*. Their positivity was also reflected in their interactions with their peers, as Julia explained, *“ I’m glad that you came to seek help rather than suffering on your own … and let us take care of you ”*. Julia found it helpful to positively reframe and reassure peers to encourage them in their recovery as peers often had negative and unpleasant past experiences in seeking treatment for their mental health problems. For their peers, participants saw the PSS role as embodying principles of connectedness, non-judgement and hope.

#### Recovery is reinforced

All participants made sense of the role they played by seeing providing peer support as leading to perpetuating effects in recovery. Participants described the PSS role as being integral to their own ongoing recovery while providing peer support and how their sharing encouraged peers, who initially did not respond to traditional treatments, to embark on their recovery journey.

Susan acknowledged that acceptance of self and others was not an easy process even in individuals without mental health conditions. Hence, she saw the process of sharing in her PSS role as valuable in bringing about her own acceptance and eventual recovery, *“ When you can talk to other people that means you are accepting yourself already. That means accepting yourself and accepting of other people in your life and their views”*. Being more accepting then seemed to facilitate her acceptance of others.

Furthermore, participants believed that using their approach to recovery and peers’ improvements brought about changes in the perceptions of professional medical teams. From John’s perspective, *“ …As I’m sharing my recovery strategies, clients (peers) start listening and feeling better, recovery strategies that I shared gets reinforced on me and I start getting better and when the system sees both of us getting better, the system starts getting better ”*. This suggests that John sees the PSS role as having value beyond the individual interactions with peers by facilitating a more encouraging environment for recovery which eventually becomes reality.

Susan and Anna found dual meaning in positive encouragements they received from their peers; seeing it as evidence of peers’ empowerment and validation of their own progress. Susan described an interaction with a peer which evoked feelings of empowerment and encouragement from her own sharing, *“ … she was saying thank you for your encouragement because you told me that I can do it. I didn’t know that I can do it and I found a job … ”*. Hence, through the role of PSS, important elements of the recovery model like provision of hope, encouragement and empowerment enabled peers to embark on their recovery journey, thereby, accentuating the effectiveness of peer support.

### Balancing on a bridge

This superordinate theme represented the tensions experienced by PSS while performing their role. Three participants, Julia, Susan, and Anna explicitly used the term “*bridge*” in their narratives to describe how they viewed themselves as a connection between mental health professionals on one side and their peers in recovery on the other side. Being this connection often meant engaging in a careful balancing act between their own and others’ recovery and between the views of mental health professionals and their peers in recovery. They saw having to deal with challenging circumstances and making difficult decisions while providing peer support as inherent in the role. Overall, participants believed personal qualities like sensitivity and maturity, and maintaining neutrality and flexibility were important when faced with challenging situations at work. In the two subordinate themes generated, participants viewed success in the PSS role as effectively striking a balance between their personal needs and the needs of their peers and between the hesitation of others and their own eagerness to push a recovery agenda.

#### Looking after self versus others

All of the participants emphasized their awareness of limits to their psychological capacity to support peers in their role. Although they enjoyed the process of supporting another in their recovery journey, they often felt challenged and overwhelmed which contributed to emotional fatigue. Mary, Susan, and Anna shared their experiences of near relapse or relapse due to insufficient self-care. As such, participants believed it was important to value themselves before caring for others. From Susan’s experience, *“ …Awareness of ourselves is important, I think we need to do what we can do … step back, what we cannot do … self-care is very important … we have the kind of energy and the self-care part is done properly, you’re able to manage ”*.

For David self-care meant pacing himself whilst at work according to his energy level, which he believed allowed him to be effective in his role as a PSS, *“So I know my energy, when I’m most energetic early in the morning then it comes to evening when I’m most tired, I cannot continue my work because I may make mistakes.”* For Julia, self-care meant being assertive both at work and at home in order to have the capacity to perform her role as PSS,

I was able to say no to some … I think at work, there are bound to be things to say no … I cannot take any more patients for now, I cannot do this, I can’t take this media engagement, this doesn’t suit me, I will tell my boss that this is not good, I need a computer. I think being assertive helps with mental health rather than being quiet. The assertiveness goes with my family as well, sometimes I’m tired, I don’t want to go out on Saturday, I say no. I’m not going out …

(Julia)

It seems that being in the role of PSS, required participants’ constant awareness of any physical or emotional symptoms to gauge their own capacity to provide peer support, being assertive in communicating their own needs for self-care and being consistent with self-care regimes to continue providing peer support.

#### Pushing forward and pulling back

All of the participants spoke with pride about overcoming the challenges they encountered while providing peer support with care and maturity. They seemed to view managing systemic expectations of peers’ recovery and being mindful of following the pace of peers in their recovery journey as another fundamental part of the PSS role.

Anna made sense of working as a PSS by situating it within and contrasting it to a mental healthcare system which is still in the early phases of adapting towards the recovery model. Here Anna is acutely aware of the need to subtly manage and work within the constraints of the mental health system and the workplace culture, by knowing when to advocate for peers and when to step back, in order to most effectively support peers’ recovery.

*It’s so dominated by…non-recoveryperson. Ithink it’s more about the person, whether they are ready to receive us, whether they are ready for our inputs…important to have peer support specialist, especially in hospitals because it’s difficult and…the culture*

Julia’s perception of her peers as feeling traumatized by the healthcare system, and as a result of disengaging from their medical team, led her to view her role as a *“bridge”* to recovery which often required her to stay neutral when supporting her peers.

I have to explain that this is the rationale, but also emphasize that they (doctors) might not be totally right … but if you really want to let them (doctors) know how you feel, I advocate the patients to go and tell them (doctors) … just talk to them (doctors), trust them (doctors), tell them (doctors) how you feel …

(Julia).

Participants saw it as their responsibility to persist in the face of rejection by peers and/or peers’ family members. They used their lived experience and sensitivity to peers’ feelings to mentally prepare themselves for negative reactions and interpreted such rejections objectively and as part of the recovery journey. David believed that PSS need to recognize peers’ readiness and acceptance towards peer support and recovery using empathy, sensitivity, and maturity to create a safe space for their peers, *“ Not everyone will buy in what you want to share with them, at that moment. And not everyone will learn and receive the message at the same time. So the aha moment takes time.”*

In addition, a few of the participants believed it was important to be observant and considerate to individual needs to tailor the peer support that would be helpful for a particular peer. Julia elaborated on her process of individualizing peer support, *“I have to think very carefully before I talk because I’m not just, I’m not just me anymore … I have to be sensitive, I need to understand how to help that person in the best way possible because everyone is different … ”*. This suggests that Julia understood the need for individualization of peer support whilst also, perhaps, feeling compelled to fulfil the image peers may have of her. Her repetition of the phrase *“have to”* indicates that she thinks she must be intentionally more aware, sensitive, and understanding to the needs of each individual peer.

### Impossible without support

This superordinate theme encapsulated the view that being effective in the PSS role was dependent on support from others. All the participants shared in detail the significance of emotional support and self-care that enabled them to perform their role as PSS. This suggests that participants perceive providing peer support as requiring immense amount of emotional strength and energy, and they recharge by receiving support from their own social support network. Three subordinate themes were generated.

#### Family support

All participants' narratives reflected the importance of receiving familial support while performing their role as PSS. They shared that the role of PSS was emotionally taxing and that their family members were understanding and provided the support needed at home. John, Julia, and Mary shared similar experiences, that there was implicit understanding from their family members without having to share with them details of their work as their family members were more concerned about their psychological well-being.

John shared that having a close relationship with his father enabled his father to be sensitive and notice any symptoms John may be experiencing. This close social support helped to detect early changes in John’s mental state which reduced John’s chances of relapsing and thus John could continue to perform his role as PSS *“ My dad is aware of it (relapse prevention plan) … so sometimes if I miss the symptoms … my dad will know.”*

Julia viewed her family’s unconditional support as essential to being able to function in the PSS role, *“my family’s understanding … I need that space, I need that time to recuperate ”*. For Julia, her family was able to step in with chores and caregiving needs while she performed her daily routine of meditation as part of self-care. Her family’s support enabled her to fulfil both her personal commitments at home and her role as a PSS. Hence, these participants believed that when their families tried to create a stress-free environment for them at home, they were able to recuperate at home which enabled them to be at their best whilst at work.

Susan saw the support from her family as giving her strength to continue in the role of PSS, *“My mother is the main support … she is a strong woman … so she gives me the kind of strength to move on … there are so many people for you to help”*. Support from the family came in different forms in each participants’ family, such as being aware of relapse symptoms, giving space whilst at home, and being the source of encouragement. These elements of unwavering and unconditional support from their families enabled the participants to gain strength, both physically and psychologically, and seemed to be essential to continuing to provide peer support to their peers.

In contrast, Anna and Julia saw the role as creating some tension with their families, mainly with media engagement and stresses faced at work. All participants were living with their family, a typical living arrangement in Singapore. Hence, ensuring peace and harmony within the family was seen as important. Media engagement often required PSS to appear on various media platforms to share their recovery journey, this also meant that PSS were required to disclose their diagnosis on media. Anna shared her struggle with her family members to seek their approval to appear on media, *“My mother is actually very supportive … my younger sister is a bit more reserved. So I try to manage”*. Anna understood that her self-disclosure of mental illness on media was associated with experiences of stigma by her family members. Ultimately the differing levels of support and acceptance within her family led Anna to take into consideration her family members’ feelings and she eventually compromised by appearing on selected types and genres of media.

Julia was similarly tolerant towards her family’s reluctance for her to appear on media, likely viewing it as reflective of widespread stigmatizing attitudes towards mental health problems in Singapore, and so she negotiated with her mum about an appropriate level of disclosure. This indicated that neither Anna nor Julia saw their role as PSS as an entirely individual pursuit.

I think my parents and my sister and brother-in-law, I mean they. They’re worried lah. Especially my mum is like you know, don’t tell people … they (media engagement) are not talking about my personal life, they (media engagement) are talking about my disorder, it’s a stigma for example … I thought that is manageable because I do tell people about my bipolar disorder and um, so I told my mum, my mum was reluctant but we came to this thing, as long as I don’t say too much about myself right, it’s fine right, and she said yeah yeah it’s fine

(Julia)

Whilst the PSS role appeared to be dependent on support some had to negotiate for the support their needed. These participants had to manage their families concern that the work was too emotionally intense for them, and their fears of possible relapse.

My mum is worried … are you being stressed back, is it affecting your health? The last thing they want is for me to get into episodes again lah, then I have to tell them it’s okay lah, you know I have learnt through my experience that I tend to make it balance … *(Julia)*

Whereas Julia appeared to be accepting of her mum’s worry, Anna viewed it as *“stifling”* as her family tended to be *“too protective”* whenever she was unwell. Anna recalled that a recent relapse at work became a turning point for her family to understand what she needed and contributed to her getting back on her feet and back to work again.

I think my family has realized how important it is to have wellness. Because it’s really difficult to go through a relapse, it’s really quite horrible, but at the same time I think my family sees me what I need as an adult person, rather than the old me who was like a teenager, like years and years ago. So I think there’s this mutual understanding.

(Anna)

Hence to perform in the PSS role these participants had to reassure their families by openly communicating their needs, such as time needed for self-care and informing families of their safety plans. With effective communication, participants were able to understand their families’ worries and address them adequately. Gaining their family members’ trust and seeing that they had faith in their ability to handle the demands of the role supports the idea that the PSS role was seen in the context of the familial system.

#### Professional support

Overall, the participants believed that professional support was valuable and necessary to their role, and this support was evaluated in terms of acceptance of recovery principles. In all the participants’ narratives, they shared that their supervisors did not have a lived experience and were trained in various mental health professions but yet they understood the recovery model and the roles of PSS in the mental health field. Their supervisors’ openness and professionalism was beneficial in providing adequate support for the participants to perform their roles as PSS. John explained, *“ I am quite fortunate because my supervisor has gone through the recovery training … she has a good idea”*. John’s supervisor, who is a non-peer professional, was able to provide adequate supervision support to enhance his learning from peer trainings received. Mary also shared how her supervisors gave her time to rest and recover when she experienced a relapse while on the job as a PSS, *“ You better rest. When you fully recover then come back ”*. For Mary, the assurance and support from her supervisors allowed her to recuperate without feeling stressed about having to take time off from work.

Anna seemed almost surprised at the success of her transition from a peer (receiving treatment) to a colleague within the same department. Though some of her colleagues were still adapting to Anna’s transition she felt that, *“ My department they are very open and actually quite well-versed in recovery principles, colleagues are very supportive”*. Another participant, David, saw collaboration within the interdisciplinary professional team and mutual contribution as leading to positive growth, *“ We meet once a week and share growth orientation with one another in the interdisciplinary team … everyone needs to know everyone’s work”*. This suggests that an inclusive work environment allowed participants to feel supported and grow in their role of PSS.

#### Faith

In addition to the support received from both home and work, the majority of the participants viewed their faith as giving them strength for their role as PSS, as Julia described *“keeps me sane”*. David also explained gaining his source of motivation and strength from his faith during difficult moments, *“ My faith, Jesus is my source of support for me, … kept me going … not looking at my own shortcomings but I can walk on water … that I can do the impossible”*. Two of the participants shared using faith to keep them safe and alleviate symptoms experienced. For John, his reliance on his faith was important in his ongoing recovery journey, *“ Reoccurrence of the mental illness … pain is really quite bad … all the recovery strategies, all the support system might not be that helpful … what keeps me safe is my spiritual beliefs”*. Overall, from participants’ experiences, having faith provided the spiritual healing, strength and assurance needed when feeling despair to persevere in difficult situations.

### Helping to end stigma

This superordinate theme describes the responsibility the participants felt in their PSS role to reduce stigma and promote the recovery approach to the extent that they willingly and repeatedly used themselves and their stories to attempt to change mindsets. On the one hand participants appeared to be proud of their personal contributions towards breaking down stigma, and on the other hand they portrayed a sense of powerless in the face of organizational, structural, and societal barriers to a more widespread adoption of the recovery approach. Two subordinate themes were generated.

#### Using self as tool

The majority of the participants saw using their lived experiences and ongoing recovery as crucial to changing the mindsets of others (e.g., medical professionals and peers) that recovery is possible and achievable. Some participants believed that medical professionals often see peers in their acute state and hence had the mindset that recovery is impossible, or that recovery only equates to a decrease in psychiatric symptoms. Julia used her interactions with other seemingly disbelieving medical professional staff to elaborate, *“when they heard that I’m a peer support specialist, they will ask … what’s your diagnosis … they have been working in the mental health industry for like 20 years or 15 years and they’ll ask me like so, are you really well?”*. Julia did not view these experiences negatively, instead, she saw them as opportunities to use her lived experience as a tool to bring about mutuality with peers and to normalize mental health diagnoses, *“ I’m actually helping to end the stigma against themselves … they are not compliant … because they reject that label … I always advocate bipolar disorder is just like oh, I am blood type O ”*.

Susan’s visceral description of her lived experience in the role of PSS gave her hope for others and courage to change mindsets of others, *“ I have tasted recovery, I feel that recovery is possible so want to spread the message to other person that we can recover ”*. Whilst John used language deliberately to attempt to challenge stigma and breakdown power hierarchies inherent within mental health services *“ Peers … That means the patients, I mean I don’t call patients as patients, I call them as peers, yeah. So I think that is the main differential one ”*.

In the process of providing peer support, all the participants used their personal definition of recovery to guide peers towards recovery. It was unanimous that the participants viewed recovery as leading their lives to the fullest. None of the participants used psychiatric symptoms to define their recovery. Hence, embracing such mindsets, participants hoped that through their interactions and personal sharing with peers and non-peer professionals, they could reduce peers’ own self-stigma and educate non-peer professionals about recovery.

Anna shared her thoughts about how she viewed her role as a catalyst to reduce stigma by creating a strong recovery culture in the mental health system and having the inner strength to withstand negativity and criticisms in the process, *“ Peer support is one of the main stewards having a recovery-oriented community because of what we have experienced, … gives the more traditional professionals an insight that they perhaps previously don’t have ”*. For Anna this appeared to be underpinned by an acute awareness of the subtle ways in which prejudice might be enacted, “ *I’m more sensitive perhaps to stigma and things like that. When I’ll feel like they are treating me a bit differently from all my co-workers and I’ll feel like oh why are you doing this special treatment ”.*

David, despite being emotionally hurt by the words of other peers which made him question his ability as a PSS *“ You think you’re peer specialist, is it? You think you are counselor is it? You are also a patient like me ”* was able to overcome this prejudice by drawing on encouragement from others who saw his potential as a PSS *“ The next thing that keep me going is the support system, the core people who believe in me, that I can contribute more, to go beyond from asking for welfare, to being able to fare well in the community ”*. He may unconsciously have felt that he needed to put his words in action in his role as PSS and be the living testimony that *“there is life after diagnosis”* which motivated him continue to reach out to other peers despite experiencing rejection.

#### One voice in the system

All the participants recognized that supportive work environments and having colleagues and supervisors who accepted the recovery model framework enabled a smooth transition into the role of PSS. However, they believed that the Singapore mental health system has not yet fully embraced the recovery model approach, thus resulting in medical professionals still operating within a traditional medicalized framework, and they seemed to feel a sense of powerlessness in their ability to effect change. Despite this, both Julia and John continued to view their contribution as an important component of eventual change.

From Julia’s perspective, *“ Hospital policy and I understand they have to go through a particular protocol and they can’t break that, it’s not something we can break … but sometimes I feel like there’s nothing much we can do lah, I can only voice out”*. Here, John described his experience during a multi-disciplinary team meeting, *“ We just advocate to the doctors that the peer is getting better … at the end of the day how the hospital runs is the (doctor) one that is more responsible for the patients ”*. These experiences suggest that participants felt limited in their role as they were not able to implement changes at systemic levels although they may be able to advocate for changes at individual or departmental levels.

With the limitations of their roles, some participants envisioned career progression to impact changes at societal or political level. David elaborates, *“ At the hospital I find that many of our peers could experience revolving doors, after their discharge then they come back to the hospital … if only the funding structure of healthcare could improve to promote more recovery-oriented services”*. John also shared his visions of societal acceptance of recovery, *“The only thing is having a good recovery culture and for that you need everyone in Singapore to get involved actually. It can’t be done by just a few people”*. Participants believed that the process of bringing about changes requires collective efforts from both top-down and bottom-up approaches to embrace the recovery model framework and therefore that the role of PSS is limited in its ability to effect change in isolation.

## Discussion

This interpretative phenomenological study aimed to explore the lived experiences of PSS and their perceptions of their role working in mental health organizations in Singapore. Previous qualitative studies involving PSS, including the only known study in Singapore, have tended to focus on broad issues such as facilitators and barriers to PSS work and benefits of and challenges in PSS work (Kuek et al., 2021; Tse et al., 2017, 2019). Whilst some of these past studies have captured elements of the PSS experience, this IPA study is, to the authors’ knowledge, one of two, and the first in Asia, to intentionally focus on the lived experiences of PSS (Dyble et al., 2014).

Participants’ experiences were encapsulated under four superordinate themes: embracing and embodying recovery, balancing on a bridge, impossible without support, and helping to end stigma. The findings also suggested, at subordinate levels, personal qualities and social and cultural factors that enabled them to face the challenges in their role, and their hopes for organizational and societal attitudinal shifts. These themes strengthen and deepen our understanding of how PSS in Singapore perceive and make sense of their roles in an environment that has been slowly shifting towards more recovery-oriented mental health services (Gun & Leong, 2016) but where stigmatizing attitudes towards mental health problems continue to exist (Pang et al., 2017; Yuan et al., 2016, 2017).

Participants’ perceptions of their PSS role were in line CHIME processes of recovery (van Weeghel et al., 2019) in that whilst CHIME processes were evident in their own recovery journeys, they were also mirrored in the peer support work they offered. They viewed connection as a central component of their work, particularly being able to connect in a sensitive and timely manner and they saw a main function of their role as bringing hope and optimism to their peers. They took on the PSS role as part of their identities and relied on their positive sense-of-self to persevere and handle challenges. They embodied recovery principles within their workplaces, whereby the PSS role became a large component of the meaning they found in life. Finally, they used their own sense of empowerment to continually offer a different message of strength to that of the traditional medicalized views of mental illness.

Similarly, the results could be interpreted in relation to the five-stage model of recovery proposed by Andresen and colleagues (2003). This theoretical framework facilitates understanding of participants’ current stage of recovery and how it enables them in their role of PSS. Participants’ narratives depicted their journeys through the different stages, and they were most likely to be at the final stage (growth). At this stage an individual is aware of their psychiatric symptoms and has developed their personal ways to manage them. They show resilience in facing the challenges at work, whilst maintaining a positive attitude, and reframing their negative experiences more positively.

Whilst not explicitly referring to use of these stages in their work, participants showed an intuitive awareness of the likely stage their peers were in. For example, they knew to approach respectfully and cautiously when peers were in moratorium stage, and how to find the right moment of awareness to share their own stories. They repeatedly used their own experiences to assist their peers with preparation. They fought against stigma encountered and advocated for the needs of themselves and their peers, embodying the stage of rebuilding for themselves as individuals, their peers, and the healthcare system. They modelled growth in the dual management of their work and their own mental health and the meaning they were able to find in the PSS role.

In line with previous findings, the themes richly describe the process by which PSS in Singapore established their role as a PSS and how the role helps to reduce self-stigma and increase self-acceptance, and to improve confidence and self-esteem (Johnson et al., 2014; Repper & Carter, 2011). The nature of the PSS work then perpetuates their recovery further as illustrated in previous research (Dyble et al., 2014; Repper & Carter, 2011; Walker & Bryant, 2013) and fitting with the mechanism of “the helper role” described by Watson (2017) in her scoping review. These similarities are important to note as they provide cross-cultural support for findings that have previously been limited to Western English-speaking studies. They also confirm the value of the PSS role in supporting self-recovery within a mental healthcare system that has recently started to shift away from institutionalized care towards more community services (Kua & Rathi, 2019) and a country that is working towards its first workplace anti-discrimination laws (Tripartite Alliance for Fair & Progressive Employment Practices, 2017).

Unlike previous research, which has often emphasized a lack of clarity around the PSS role and a lack of support from colleagues (Kuek et al., 2021; Mutschler et al., 2021), participants in this study were largely positive about the support they received. They described their supervisors as supportive and professionals generally as being open-minded towards accepting a recovery-oriented approach despite their various training specializations. This could be due to the difference in research aims, in that previous studies specifically focused on barriers to peer support implementation amongst professionals. In contrast, participants in this study believed that peers were less attuned to the recovery model as compared to non-peer professionals. As a result of this, participants tried to individualize their recovery stories to share during peer support to encourage hope and empower peers in their own recovery journey as suggested by Watson (2017).

Building on findings about the stressful nature of the PSS role (Moran et al., 2013; Repper & Carter, 2011) and the need to identify personal coping strategies (Kuek et al., 2021), this study provided rich examples of internal cognitive, emotional and spiritual processes that participants went through in order to balance their own needs with the needs of their peers. The importance given to self-care, with examples of particular coping strategies, helps to elucidate the nuances of a role that requires individuals to use deeply personal experiences within a professional setting. For example, self-awareness was used as a tool for managing both personal stress levels and building rapport with peers via stories of struggle and hope.

Family support, in particular, was identified as a coping resource. The importance of family support is an aspect that appears to be unique to a non-Western context—it was also emphasized in Tse et al. (2017) - that enabled participants to perform in their role as PSS. Family support is not mentioned by Dyble et al. (2014) or Ahmed et al. (2015) and support in general is mentioned only briefly by Clossey et al. (2016) and Moran et al. (2013). Findings from this study emphasize the importance of the family system in Singapore, both in terms of the value of support family members can provide and the influence of their views. Participants’ families had an implicit understanding of participants’ needs and continued to provide their support, albeit sometimes conditionally. This implicit understanding facilitated participants’ ability to self-care and reduced additional stress from family. In contrast, participants in Western studies tend not to report the importance of family support enabling them in their role of PSS focusing more on organizational and/or supervisorial support (e.g., Clossey et al., 2016; Dyble et al., 2014).

The individualism-collectivism (IC) paradigm is helpful to explain participants’ experiences of providing peer support and the ways in which family support is primarily helpful, though occasionally harmful (Tse & Ng, 2014). The IC paradigm highlights the existence of both vertical dimensions of hierarchical structure and horizontal dimensions of importance of equality within a context. This concept summarizes participants’ experiences of collectivist values that promote recovery (e.g., provision of family support) and individualism values that participants take control of their recovery journey (e.g., prioritizing self-care strategies). The collectivist values that hindered recovery in participants include overprotection from family and concerns about the impact of an individual on the family unit (e.g., seeking approval for media advocacy).

This finding in particular, along with the value placed on faith by the participants, offers support for Kuek et al. (2021) and van Weegehel et al.’s (2019) recommendation that paying attention to relevant personal, social and spiritual factors related to recovery is imperative to understanding cultural differences in recovery. In Singapore it seems that assuming the identity of a PSS, whilst offering meaning and empowerment to the individual, has implications for the family system. As such, the PSS role is concurrently seen through the individual’s eyes and those of the family, and the individual’s level of immersion in the role sometimes has to be negotiated with the family.

Finally, the findings highlighted the dominance of stigma across various levels: individual, family, and systemic. Participants valued their PSS role in reducing and minimizing stigma associated with mental illness and recovery. From participants’ accounts, stigma towards mental illness is engrained in Singapore’s society, as both peers and non-peer professionals continue to adopt the medical model of recovery and understanding of mental illness similar to a previous Singapore study (Kuek et al., 2020), and to Western studies (e.g., Kemp & Henderson, 2012). However, in Singapore, the magnitude of stigma is greater as the recovery framework is in an earlier stage of implementation and adoption (Kuek et al., 2020; Yuan et al., 2016, 2017).

At the individual level, the superordinate theme of balancing on a bridge suggests that stigma is largely associated with participants’ and peers’ psychiatric symptoms, suggesting an internalization of the medical model. Participants then had to work hard in the PSS role to engage and earn the trust of their peers and their peers’ families who likely have a similarly traditional view of recovery, and they had to know when to step back from more forthright advocacy. The PSS role in itself helps to challenge the dominant medical model understanding of recovery as do the individual contributions of PSS (Mahlke et al., 2014).

At the family level, the subordinate theme of family support suggests that stigma is significant within participants’ families due to the tension experienced. The framework of family recovery conceptualized by Spaniol (2010) explains the impact experienced by family members after the onset of mental illness in their loved ones, namely: shock, discovery, and denial; recognition and acceptance; coping; personal and political advocacy. Family members experience stigma similar to that experienced by individuals with mental illness. They may also internalize the stigma surrounding mental illness, leading to self-blame, guilt, and maintaining secrecy about the mental health condition of their loved ones (Spaniol & Nelson, 2015). This may be further exacerbated in Asian families who may experience face concern in relation to a family member’s mental illness, particularly if they were to publicly identify themselves via media engagements (Chen et al., 2020; Ho, 1976).

At the systemic level, the superordinate theme of one voice in the system suggests participants’ limitations in advocating for peers within the traditional medical mental health system which in turn may have negatively impacted their role as PSS. Similar to Western findings (Clossey et al., 2016; Mutschler et al., 2021), the medical model poses a barrier for successful implementation of peer support. Therefore, with PSS’ unwavering efforts to continue to voice their struggles in their role, their participation may contribute to changes at systemic levels (Shera & Ramon, 2013).

## Clinical and research implications

This study contributes to the PSS literature by giving voice to the experience of PSS working in mental health organizations in Singapore and hearing their perspectives on what it is like to be a PSS in the mental healthcare system. First, the participants’ narratives in this study unanimously testify to the value of the PSS role for individuals and their peers, as well as to their potential for breaking down stigma and facilitating adoption of more recovery-oriented mindsets amongst professionals and organizations. However, challenging the continued existence of stigma at various levels within the healthcare system and wider community must not fall to PSS alone, it must be challenged at every level within society. More could be done to challenge stigma within the community, and the effectiveness of these programmes to reduce stigma must be evaluated (Kuek et al., 2020). Changes must also occur from top-down, whereby changes in mental healthcare systems (i.e., being less hierarchical) and training and familiarizing more staff with recovery approaches occur in parallel with social justice movements. This will ensure the founding principles of peer support delivery are adhered to (Stratford et al., 2017).

Second, the importance placed on family support and the potentially hindering influence of family in this study suggests the need to take account of family systems and family attitudes when recruiting, training and supporting PSS at work. Third, theoretically, the findings from this research are in line with existing models of recovery that consider both the processes (van Weeghel et al., 2019) and stages of recovery (Andresen et al., 2003). The PSS role is perceived by those that do it as embodying the processes and reflecting the stages of the recovery journey. It would be interesting and helpful to understand whether organizations and the healthcare systems they sit within can similarly embody these stages. There is much written about how, and how effectively, organizations and services implement recovery-oriented approaches and to some extent whether they operate in line with recovery principles. There is not enough about what stage of recovery these organizations and systems are in; are they still in a state of denial and hopelessness or are they starting to become aware of and hopeful about their own recovery and preparing themselves for change and putting the required effort into rebuilding?

In terms of future research, to have a better understanding about PSS in Singapore, it would be essential to explore areas like effectiveness of peer support in Singapore and understanding attitudes towards recovery in mental health (non-peer) professionals and family members.

## Limitations

A potential limitation of this research is the homogeneity of the sample in terms of their organizations. Most of the participants worked within the same context; therefore, the depth and diversity of experiences of PSS population in Singapore may not be reflected in this sample. Another possible limitation of this study is the first author’s background in working with PSS, however this insider-researcher perspective could also be considered a strength given her ability to engage with the participants and to analyse the results through the lens of professional experience (Taylor, 2011). Finally, it is acknowledged that in our attempt to ask about multiple aspects of the PSS experience to fully understand how PSS viewed their role we may have sacrificed depth of understanding over breadth (Pietkiewicz & Smith, 2014).

## Conclusion

This study contributes to the PSS literature in a non-Western context, to understand how participants perceive their roles as PSS within the mental healthcare system. Overall, the findings suggest that participants perceive the role as beneficial to their own recovery in terms of connection, hope, identity, meaning and empowerment, thus aligning with existing models of recovery. They perceive it as valuable in supporting their peers practically and supporting the development of recovery-oriented mindsets. Finally, they perceive it as having manageable challenges, provided they attend to their own mental health needs and have access to different kinds of support. A notable result from this study is the valued and complex aspect of family support, similar to results in another non-Western study (Tse et al., 2017), which might be unique to a non-Western or collectivist culture, where the family concurrently offers support and asks for conformity. Overall, PSS in Singapore make sense of their role at multiple levels, through the eyes of their peers, family members and professionals they work with and perceive it as having a small, but essential, role to play in challenging stigma amongst professionals, organizations and the mental healthcare system. The part they play in breaking down stigma needs to be matched by their non-peer professionals and at organizational and societal levels.

## References

[cit0001] Ahmed, A. O., Hunter, K. M., Mabe, A. P., Tucker, S. J., & Buckley, P. F. (2015). The professional experiences of peer specialists in the Georgia mental health consumer network. *Community Mental Health Journal*, 51(4), 424–15. 10.1007/s10597-015-9854-8 25724917

[cit0002] Ali, K., Farrer, L., Gulliver, A., & Griffiths, K. M. (2015). Online peer-to-peer support for young people with mental health problems: A systematic review. *JMIR Mental Health*, 2(2), e19. 10.2196/mental.4418 26543923 PMC4607385

[cit0003] Andresen, R., Oades, L., & Caputi, P. (2003). The experience of recovery from schizophrenia: Towards an empirically validated stage model. *The Australian and New Zealand Journal of Psychiatry*, 37(5), 586–594. 10.1046/j.1440-1614.2003.01234.x 14511087

[cit0004] Braun, V., & Clarke, V. (2006). Using thematic analysis in psychology. *Qualitative Research in Psychology*, 3(2), 77–101. 10.1191/1478088706qp063oa

[cit0005] Chamberlin, J. (1995). Rehabilitating ourselves: The psychiatric survivor movement. *International Journal of Mental Health*, 24(1), 39–46. 10.1080/00207411.1995.11449302

[cit0006] Chen, S. X., Mak, W. W., & Lam, B. C. (2020). Is it cultural context or cultural value? Unpackaging cultural influences on stigma toward mental illness and barrier to help-seeking. *Social Psychological and Personality Science*, 11(7), 1022–1031. 10.1177/1948550619897482

[cit0007] Clossey, L., Gillen, J., Frankel, H., & Hernandez, J. (2016). The experience of certified peer specialists in mental health. *Social Work in Mental Health*, 14(4), 408–427. 10.1080/15332985.2015.1038412

[cit0008] Cronise, R., Teixeira, C., Rogers, E. S., & Harrington, S. (2016). The peer support workforce: Results of a national survey. *Psychiatric Rehabilitation Journal*, 39(3), 211–221. 10.1037/prj0000222 27618458

[cit0009] Davidson, L., Bellamy, C., Guy, K., & Miller, R. (2012). Peer support among persons with severe mental illnesses: A review of evidence and experience. *World Psychiatry: Official Journal of the World Psychiatric Association (WPA)*, 11(2), 123–128. 10.1016/j.wpsyc.2012.05.009 22654945 PMC3363389

[cit0010] Davidson, L., Chinman, M., Kloos, B., Weingarten, R., Stayner, D., & Tebes, J. K. (1999). Peer support among individuals with severe mental illness: A review of the evidence. *Clinical Psychology: Science and Practice*, 6(2), 165–187. 10.1093/clipsy.6.2.165

[cit0011] Deegan, P. E. (2002). Recovery as a self-directed process of healing and transformation. *Occupational Therapy in Mental Health*, 17(3–4), 5–21. 10.1300/J004v17n03_02

[cit0012] Dyble, G., Tickle, A., & Collinson, C. (2014). From end user to provider: Making sense of becoming a peer support worker using interpretative phenomenological analysis. *Journal of Public Mental Health*, 13(2), 83–92. 10.1108/JPMH-03-2013-0016

[cit0013] Fortuna, K. L., Naslund, J. A., LaCroix, J. M., Bianco, C. L., Brooks, J. M., Zisman-Ilani, Y., Muralidharan, A., & Deegan, P. (2020). Digital peer support mental health interventions for people with a lived experience of a serious mental illness: Systematic review. *JMIR Mental Health*, 7(4), e16460. 10.2196/16460 32243256 PMC7165313

[cit0014] Griffiths, C. A., & Hancock-Johnson, E. (2017). The experiences of paid formal lived experience workers within a secure mental health service. *The Journal of Mental Health Training, Education and Practice*, 12(5), 313–322. 10.1108/JMHTEP-09-2016-0046

[cit0015] Gun, S. Y., & Leong, J. J. (2016). Social inclusion: The Singapore story in community mental health development, psychiatric rehabilitation and recovery. *Asia Pacific Journal of Social Work and Development*, 26(2–3), 167–177. 10.1080/02185385.2016.1218793

[cit0016] Ho, D. Y. F. (1976). On the concept of face. *The American Journal of Sociology*, 81(4), 867–884. 10.1086/226145

[cit0017] Ibrahim, N., Thompson, D., Nixdorf, R., Kalha, J., Mpango, R., Moran, G., Mueller-Stierlin, A., Ryan, G., Mahlke, C., Shamba, D., Puschner, B., Repper, J., & Slade, M. (2020). A systematic review of influences on implementation of peer support work for adults with mental health problems. *Social Psychiatry and Psychiatric Epidemiology*, 55(3), 285–293. 10.1007/s00127-019-01739-1 31177310

[cit0018] Johnson, G., Magee, C., Maru, M., Furlong-Norman, K., Rogers, E. S., & Thompson, K. (2014). Personal and societal benefits of providing peer support: A survey of peer support specialists. *Psychiatric Services*, 65(5), 678–680. 10.1176/appi.ps.201300113 24535565

[cit0019] Kemp, V., & Henderson, A. R. (2012). Challenges faced by mental health peer support workers: Peer support from the peer supporter’s point of view. *Psychiatric Rehabilitation Journal*, 35(4), 337–340. 10.2975/35.4.2012.337.340 22491374

[cit0020] Kua, E. H., & Rathi, M. (2019). Mental health care in Singapore: Current and future challenges. *Taiwanese Journal of Psychiatry*, 33(1), 6–12. 10.4103/TPSY.TPSY_2_19

[cit0021] Kuek, J., Chua, H. C., & Poremski, D. (2021). Barriers and facilitators of peer support work in a large psychiatric hospital: A thematic analysis. *General Psychiatry*, 34(3), e100521. 10.1136/gpsych-2021-100521 34222796 PMC8212403

[cit0022] Kuek, J. H. L., Raeburn, T., & Wand, T. (2020). Asian perspectives on personal recovery in mental health: A scoping review. *Journal of Mental Health*, 1–17. 10.1080/09638237.2020.1818709 32915681

[cit0023] Lapidos, A., Jester, J., Ortquist, M., Werner, P., Ruffolo, M. C., & Smith, M. (2018). Survey of peer support specialists: Professional activities, self-rated skills, job satisfaction, and financial well-being. *Psychiatric Services*, 69(12), 1264–1267. 10.1176/appi.ps.201800251 30301447 PMC12428690

[cit0024] Lawton-Smith, S. (2013). Peer support in mental health: Where are we today? *The Journal of Mental Health Training, Education and Practice*, 8(3), 152–158. 10.1108/JMHTEP-03-2013-0009

[cit0025] Leamy, M., Bird, V., Le Boutillier, C., Williams, J., & Slade, M. (2011). Conceptual framework for personal recovery in mental health: Systematic review and narrative synthesis. *The British Journal of Psychiatry: The Journal of Mental Science*, 199(6), 445–452. 10.1192/bjp.bp.110.083733 22130746

[cit0026] Lloyd-Evans, B., Mayo-Wilson, E., Harrison, B., Istead, H., Brown, E., Pilling, S., Johnson, S., & Kendall, T. (2014). A systematic review and meta-analysis of randomised controlled trials of peer support for people with severe mental illness. *BMC Psychiatry*, 14(1), Article 39. 10.1186/1471-244X-14-39 PMC3933205 24528545

[cit0027] Mahlke, C. I., Krämer, U. M., Becker, T., & Bock, T. (2014). Peer support in mental health services. *Current Opinion in Psychiatry*, 27(4), 276–281. 10.1097/YCO.0000000000000074 24852058

[cit0028] Mak, W., Fu, A., Auyeung, L., Cheng, W., Chan, R., Tse, S., Yau, S., Ho, K., Chan, S. K., & Wong, S. (2021). Nine-month longitudinal impact of peer support workers’ recovery attributes on service users’ recovery in Hong Kong. *Psychiatric Services* *(Washington, D.C.)*, 72(11), 1282–1287. 10.1176/appi.ps.202000006 34015963

[cit0029] Miyamoto, Y., & Sono, T. (2012). Lessons from peer support among individuals with mental health difficulties: A review of the literature. *Clinical Practice and Epidemiology in Mental Health: CP & EMH*, 8(1), 22–29. 10.2174/1745017901208010022 22563347 PMC3343315

[cit0030] Moran, G. S., Russinova, Z., Gidugu, V., & Gagne, C. (2013). Challenges experienced by paid peer providers in mental health recovery: A qualitative study. *Community Mental Health Journal*, 49(3), 281–291. 10.1007/s10597-012-9541-y 23117937

[cit0031] Mutschler, C., Bellamy, C., Davidson, L., Lichtenstein, S., & Kidd, S. Implementation of peer support in mental health services: A systematic review of the literature. (2021). *Psychological Services*, 19(2), 10.1037/ser0000531. Advance online publication. 10.1037/ser0000531 33793284

[cit0032] Nizza, I. E., Farr, J., & Smith, J. A. (2021). Achieving excellence in interpretative phenomenological analysis (IPA): Four markers of high quality. *Qualitative Research in Psychology*, 18(3), 1–18. 10.1080/14780887.2020.1854404

[cit0033] Pang, S., Liu, J., Mahesh, M., Chua, B. Y., Shahwan, S., Lee, S. P., Vaingankar, J. A., Abdin, E., Fung, D., Chong, S. A., & Subramaniam, M. (2017). Stigma among Singaporean youth: A cross-sectional study on adolescent attitudes towards serious mental illness and social tolerance in a multiethnic population. *BMJ Open*, 7(10), e016432. 10.1136/bmjopen-2017-016432 PMC5652546 29042379

[cit0034] Pietkiewicz, I., & Smith, J. A. (2014). A practical guide to using interpretative phenomenological analysis in qualitative research psychology. *Psychological Journal*, 20(1), 7–14. 10.14691/CPPJ.20.1.7

[cit0035] Repper, J., & Carter, T. (2011). A review of the literature on peer support in mental health services. *Journal of Public Mental Health*, 20(4), 392–411. 10.3109/09638237.2011.583947 21770786

[cit0036] Shera, W., & Ramon, S. (2013). Challenges in the implementation of recovery-oriented mental health policies and services: Analysis of developments in England and Canada. *International Journal of Mental Health*, 42(2–3), 17–42. 10.2753/IMH0020-7411420202

[cit0037] Slade, M., Amering, M., & Oades, L. (2008). Recovery: An international perspective. *Epidemiology and Psychiatric Sciences*, 17(2), 128–137. 10.1017/S1121189X00002827 18589629

[cit0038] Smith, J. A. (2011). Evaluating the contribution of interpretative phenomenological analysis. *Health Psychology Review*, 5(1), 9–27. 10.1080/17437199.2010.510659

[cit0039] Smith, J. A. (2017). Interpretative phenomenological analysis: Getting at lived experience. *The Journal of Positive Psychology*, 12(3), 303–304. 10.1080/17439760.2016.1262622

[cit0040] Smith, J. A., & Osborn, M. (2008). Interpretative phenomenological analysis. In J. Smith (Ed.), *Qualitative psychology: A practical guide to research methods* (pp. 53–80). Sage.

[cit0041] Social Service Institute. (2022). Certificate in peer support. https://www.ssi.gov.sg/training/cet-programmes/certificate-in-peer-support/

[cit0042] Spaniol, L. (2010). The pain and the possibility: The family recovery process. *Community Mental Health Journal*, 46(5), 482–485. 10.1007/s10597-010-9315-3 20464490

[cit0043] Spaniol, L., & Nelson, A. (2015). Family recovery. *Community Mental Health Journal*, 51(7), 761–767. 10.1007/s10597-015-9880-6 25947133

[cit0044] Stratford, A. C., Halpin, M., Phillips, K., Skerritt, F., Beales, A., Cheng, V., Hammond, M.,… O‘Hagan, M., Loreto, C., Tiengtom, K., Kobe, B., Harrington, S., Fisher, D., & Davidson, L. (2017). The growth of peer support: An international charter. *Journal of Mental Health*, (6), 1–6. 10.1080/09638237.2017.1340593 28682640

[cit0045] Taylor, J. (2011). The intimate insider: Negotiating the ethics of friendship when doing insider research. *Qualitative Research*, 11(1), 3–22. 10.1177/1468794110384447

[cit0046] Thorton, T., & Lucas, P. (2011). On the very idea of recovery for mental health. *Journal of Medical Ethics*, 37(1), 24–28. 10.1136/jme.2010.037234 21059632

[cit0047] Tripartite Alliance for Fair & Progressive Employment Practices. (2017). Fair and progressive practices at work. https://www.tal.sg/tafep/-/media/tal/tafep/resources/publications/files/2017/tafep-fair-and-progressive-practices-at-work.pdf

[cit0048] Tse, S., Mak, W. W. S., Lo, I. W. K., Liu, L. L., Yuen, W. W. Y., Yau, S. …Ho, K., Chan, S.K., & Wong, S. (2017). A one-year longitudinal qualitative study of peer support services in a non-western context: The perspectives of peer support workers, service users, and co-workers. *Psychiatry Research*, 255, 27–35. 10.1016/j.psychres.2017.05.007 28511051

[cit0049] Tse, S., & Ng, R. M. K. (2014). Applying a mental health recovery approach for people from diverse backgrounds: The case of collectivism and individualism paradigms. *Journal of Psychosocial Rehabilitation and Mental Health*, 1(1), 7–13. 10.1007/s40737-014-0010-5

[cit0050] Tse, S., Siu, B. W. M., & Kan, A. (2013). Can recovery-oriented mental health services be created in Hong Kong? struggles and strategies. *Administration and Policy in Mental Health and Mental Health Services Research*, 40(3), 155–158. 10.1007/s10488-011-0391-7 22160805 PMC3616219

[cit0051] Tse, S., Yuen, W. W. Y., Murray, G., Davidson, L., Lai, Q., & Kan, A. (2019). Combining technical and expert-by-experience knowledge in the quest for personal recovery from bipolar disorder: A qualitative study. *BMC Psychiatry*, 19(1), Article 368. 10.1186/s12888-019-2357-3 31771532 PMC6878707

[cit0052] Vandewalle, J., Debyser, B., Beeckman, D., Vandecasteele, T., Van Hecke, A., & Verhaeghe, S. (2016). Peer workers’ perceptions and experiences of barriers to implementation of peer worker roles in mental health services: A literature review. *International Journal of Nursing Studies*, 60, 234–250. 10.1016/j.ijnurstu.2016.04.018 27297384

[cit0053] van Weeghel, J., van Zelst, C., Boertien, D., & Hasson-Ohayon, I. (2019). Conceptualizations, assessments, and implications of personal recovery in mental illness: A scoping review of systematic reviews and meta-analyses. *Psychiatric Rehabilitation Journal*, 42(2), 169–181. 10.1037/prj0000356 30843721

[cit0054] Walker, G., & Bryant, W. (2013). Peer support in adult mental health services: A metasynthesis of qualitative findings. *Psychiatric Rehabilitation Journal*, 36(1), 28–34. 10.1037/h0094744 23477647

[cit0055] Watson, E. (2017). The mechanisms underpinning peer support: A literature review. *Journal of Mental Health (Abingdon, England)*, 28(6), 677–688. 10.1080/09638237.2017.1417559 29260930

[cit0056] White, S., Foster, R., Marks, J., Morshead, R., Goldsmith, L., Barlow, S., Sin, J., & Gillard, S. (2020). The effectiveness of one-to-one peer support in mental health services: A systematic review and meta-analysis. *BMC Psychiatry*, 20(1), Article 534. 10.1186/s12888-020-02923-3 33176729 PMC7657356

[cit0057] Whitley, R., & Drake, R. E. (2010). Recovery: A dimensional approach. *Psychiatric Services*, 61(12), 1248–1250. 10.1176/ps.2010.61.12.1248 21123410

[cit0058] Yardley, L. (2000). Dilemmas in qualitative health research. *Psychology & Health*, 15(2), 215–228. 10.1080/08870440008400302

[cit0059] Yuan, Q., Abdin, E., Picco, L., Vaingankar, J. A., Shahwan, S., Jeyagurunathan, A.… Sagayadevan, V., Shafie, S., Tay, J., Chong, S.A., & Subramaniam, M. (2016). Attitudes to mental illness and its demographic correlates among general population in Singapore. *Plos One*, 11(11), e0167297. 10.1371/journal.pone.0167297 27893796 PMC5125689

[cit0060] Yuan, Q., Picco, L., Chang, S., Abdin, E., Chua, B. Y., Ong, S.… Yow, K.L., Chong, S.A., & Subramaniam, M. (2017). Attitudes to mental illness among mental health professionals in Singapore and comparisons with the general population. *Plos One*, 12(11), e0187593. 10.1371/journal.pone.0187593 29145419 PMC5690645

[cit0061] Yuen, W. W., Tse, S., Murray, G., & Davidson, L. (2019). ‘From my point of view, my wife has recovered’: A qualitative investigation of caregivers’ perceptions of recovery and peer support services for people with bipolar disorder in a Chinese community. *The International Journal of Social Psychiatry*, 65(4), 305–312. 10.1177/0020764019842287 30983474

